# Report on Pairing-based Cryptography

**DOI:** 10.6028/jres.120.002

**Published:** 2015-02-03

**Authors:** Dustin Moody, Rene Peralta, Ray Perlner, Andrew Regenscheid, Allen Roginsky, Lily Chen

**Affiliations:** National Institute of Standards and Technology, Gaithersburg, MD 20899

**Keywords:** IBE, identity-based encryption, pairing-based cryptography, pairings

## Abstract

This report summarizes study results on pairing-based cryptography. The main purpose of the study is to form NIST’s position on standardizing and recommending pairing-based cryptography schemes currently published in research literature and standardized in other standard bodies.

The report reviews the mathematical background of pairings. This includes topics such as pairing-friendly elliptic curves and how to compute various pairings. It includes a brief introduction to existing identity-based encryption (IBE) schemes and other cryptographic schemes using pairing technology. The report provides a complete study of the current status of standard activities on pairing-based cryptographic schemes. It explores different application scenarios for pairing-based cryptography schemes. As an important aspect of adopting pairing-based schemes, the report also considers the challenges inherent in validation testing of cryptographic algorithms and modules.

Based on the study, the report suggests an approach for including pairing-based cryptography schemes in the NIST cryptographic toolkit. The report also outlines several questions that will require further study if this approach is followed.

## 1. Introduction

Recently, pairings on elliptic curves have been a very active area of research in cryptography. Pairings map pairs of points on an elliptic curve into the multiplicative group of a finite field. Their unique properties have enabled many new cryptographic protocols that had not previously been feasible.

The first use of pairings in cryptography was in 1991, when they were used to attack certain elliptic curve cryptosystems that used *supersingular elliptic curves*. Ten years later, three seminal papers used pairings in a constructive manner to implement novel (or vastly improved) protocols: Boneh and Franklin’s identity-based encryption scheme [1], Boneh, Lynn, and Schacham’s short signature scheme [2], and Joux’s one round tripartite key exchange [3]. Their work jump-started interest in pairing-based cryptography, which has grown exponentially since then. By 2004, there were already over 200 articles published on this topic, and the number today is in the thousands.

In particular, identity-based encryption (IBE) has received much attention. With traditional public key cryptography, if a user, Bob, wishes to send a message to another user, Alice, he needs to know Alice’s public key. How can Bob be sure he has Alice’s key, and not some other key substituted by a malicious attacker? There are ways to authenticate Alice’s key using certificates, but in 1984 Shamir proposed the idea of using some form of Alice’s identity for her public key [4]. This could be her email address, for instance. Anyone sending a message to Alice would then know Alice’s public key. One of the main advantages of IBE is that it allows a user to encrypt a message without needing keys to have been previously distributed. Such a scenario is quite useful if pre-distribution of keys is impractical. It was an open problem to devise an efficient IBE scheme until Boneh and Franklin’s paper in 2001.

Besides IBE, there are a number of other applications of pairing-based cryptography. These include many identity-based cryptosystems (including signature schemes), key establishment schemes, functional (or attribute-based) encryption, and privacy-enhancing techniques such as anonymous credentials.

Pairing-based cryptography has been adopted commercially. The two largest companies in this field are Voltage Security (co-founded by Boneh), and Trend Micro. In 2008, the National Institute of Standards and Technology (NIST) held a workshop on pairing-based cryptography. Over 80 people from academia, government and industry attended. Dr. Matt Franklin, co-inventor of one of the first practical IBE schemes, presented a keynote address on IBE. The second keynote address was delivered by Brent Waters, who introduced an application of pairing-based cryptography called *functional encryption*. Functional encryption allows anyone that possesses a particular set of attributes, which are defined at the time the message is encrypted, to decrypt the message.

The workshop program included presentations on new applications of pairing-based cryptography. Several presenters described how IBE and other pairing-based cryptography schemes were being used in pilot projects and live systems. The workshop also had two panel discussions. The first panel discussed where and how IBE could fit into the NIST cryptographic toolkit, along with barriers to adoption. This panel included representatives from industry, standards organizations and users. The second panel discussed IBE’s relative strengths and weaknesses compared to traditional public key cryptography, and included panelists with expertise in IBE and public key infrastructure (PKI).

The interest in pairings that was evident at the workshop has resulted in efforts to standardize pairing-based protocols. Indeed, the Internet Engineering Task Force (IETF) has started developing certain standards for some pairing-based cryptosystems. The Institute of Electrical and Electronics Engineers (IEEE) is also currently developing similar standards, as is the International Standards Organization (ISO).

Throughout the past year, the authors have been meeting to help determine whether or not NIST should begin any standardization. The distinctive properties of pairings enable cryptographic schemes with compelling features, such as IBE. Extensive research must be undertaken before NIST makes recommendations for the use of pairing technologies within the federal government. In this document, we summarize what we have learned, and share our findings.

We first give a brief, high-level mathematical background for pairings. More detail can be found in the Appendix. This background will be followed by a discussion of the most important pairing-based protocols. This discussion includes examples of IBE, signature schemes, and key establishment mechanisms. We also survey which pairing-based cryptosystems are being used commercially, as well as potential future uses. We then review the status of pairings in other industry standards. We end with our recommendations.

## 2. Mathematical Background

Since 1985, elliptic curves have been used in cryptography. Elliptic curve cryptosystems have some advantages over other systems. For example, there is a wide range of parameters for a user to choose from. Given any prime power *q* = *p^f^*, (*p* ≠ 2,3), we can define an elliptic curve over *F_q_* by the equation

$$E:y^{2}=x^{3}+ax+b$$ (1)

where *a* and *b* in *F_q_* are chosen so that 4*a*^3^ + 27*b*^2^ ≠ 0. More importantly, elliptic curve cryptosystems can offer the same level of security as other systems with a much smaller key length. This property has made elliptic curve cryptography an increasingly popular choice. NIST has recommended fifteen specific elliptic curves for government use in cryptographic applications, and NIST has standardized the elliptic curve digital signature algorithm (ECDSA), as well as the Diffie-Hellman and MQV key agreement schemes.

A point on the elliptic curve *E* is represented as an ordered pair (*x*, *y*) satisfying Eq. (1). The coordinates *x* and *y* are elements of the finite field *F_q_*. There is a way to “add” two points on an elliptic curve, and always get another point. There is also an additional, special point denoted by ∞. The defining property of the point ∞ is that if *P* is any point on the curve *E*, then *P* + ∞ = *P*.

A pairing is a function *e* that takes a pair of two points on an elliptic curve and outputs an element in a finite field. The pairings we consider are also bilinear. This means that the pairing map preserves the additive structure of the elliptic curve, and carries it over into the finite field. For example,

$$e(P_{1}+P_{2},Q)=e(P_{1},Q)*e(P_{2},Q),$$

where the * denotes multiplication in the finite field. This bilinearity property has enabled the construction of new cryptographic protocols using pairings.

Although in theory pairings exist for any elliptic curve, in practice there are curves whose pairings are not suitable for cryptographic applications. Associated to each elliptic curve, there is a parameter that can be calculated known as the *embedding degree k*. In order to efficiently implement pairings for use in cryptography, we need *k* to be relatively small, certainly less than 100. However, it has been shown that almost all elliptic curves have very large *k*. In fact, *k* is usually about the same size as *q*, which is at least 160 bits. There are two common ways to find pairing-friendly elliptic curves. The first is to use what are known as supersingular elliptic curves, which always have *k* ≤ 6. The second way is to use a technique called the *complex multiplication* (CM) method to construct certain families of elliptic curves with small *k*. There are advantages and drawbacks to each way. See [5] for a survey paper that reviews all known methods to find such pairing-friendly curves.

In order to actually implement any pairing-based cryptographic protocol, it is necessary to also choose a specific pairing function *e*. The two most commonly used pairings are the Weil and Tate pairings. With the goal of speeding up computation, researchers have discovered several new pairings. These include the Ate, Eta, reduced Tate, twisted Ate, and R-Ate pairings among others. For more information on these pairings, as well as on pairing-friendly curves, see the Appendix.

We note the various pairings are not interchangeable. For example, the Eta pairing can only be defined for supersingular curves. The Weil pairing satisfies *e*(*P*, *P*) = 1 for any point *P* in the domain, while the other pairings do not. The choice of pairing (and elliptic curve) is important. Galbraith, Patterson, and Smart, experts in this field, explain:

“Many … treat pairings as a “black box” and then proceed to build various cryptographic schemes making use of assumed properties of the pairings. This is not necessarily a bad approach, since the details of pairings, particularly their selection and implementation, can be quite complex. As an approach, it allows one to ignore mathematical and algorithmic subtleties and focus on purely cryptographic aspects of the research. However, if this approach is taken, then it is easy to make assumptions concerning the properties of pairings which are not necessarily correct, and hence develop cryptographic schemes which cannot be realized in practice, or which cannot be implemented as efficiently as the authors assume. [6]”

It is important to keep this observation in mind. However, the applications that we will discuss can all be implemented by a suitable choice of curve and pairing. We therefore will not concern ourselves with these details in the main body of this document. See the Appendix for more information on these issues.

We end this section with some remarks on the security of pairing-based cryptography. Much of elliptic curve cryptography relies on the difficulty of two problems known as the discrete log problem (DLP) and the computational Diffie-Hellman problem (CDHP). These problems have been well studied, and if parameters are correctly chosen then they are believed to provide adequate security. The security assumption behind pairing-based cryptography is known as the bilinear Diffie-Hellman problem (BDHP). This is a much newer problem, which has not been as well-studied. It is known that if one can solve the DLP or CDHP then one can also solve the BDHP. So the security of pairing-based cryptography is not stronger than that of elliptic curve cryptography. There are no currently known attacks to break the BDHP, and it is the focus of much research.

## 3. Identity-Based Encryption Schemes

IBE is similar to classical public key cryptography in that each user has a public key for encryption and a private key for decryption. However, unlike classical public key encryption, where the public key is generated from the private key, IBE allows public keys to be set to the value of a pre-existing identifier, such as an email address.

Another difference is that for IBE the individual users cannot generate their own private keys, but must instead download them from a trusted third party known as the Private Key Generator (PKG). Furthermore, in order to encrypt messages, the sender must obtain public “system parameters” from the PKG. These system parameters are used in combination with the intended recipient’s identity string to generate an encrypted message.

Thus, IBE consists of four interrelated algorithms:

- Setup – the PKG establishes the system parameters.
- Encryption – a sender uses the recipient’s identity string and the system parameters to encrypt a message.
- Private Key Generation – the PKG generates a private key for a given ID string, using the secrets established during the Setup algorithm.
- Decryption – the recipient uses a private key obtained from the PKG to decrypt a message.

While the concept of IBE was first proposed by Shamir in 1985 [4], he was only able to provide a method for a conceptually similar, but not nearly as useful, identity-based signature scheme. It was not until 20 years later that a practical scheme for IBE was actually published [1]. The tool that made the Boneh-Franklin scheme possible was bilinear pairings.

A simplified version of the Boneh-Franklin scheme works as follows:

**Setup**: The PKG chooses a pairing-friendly curve, a pairing *e*, and a base-point *P*. It then generates a secret integer, *s*. The system parameters are *P*, *sP*, *e*, and the curve.
**Encryption**: First, the sender obtains the system parameters from the PKG and converts the recipient’s ID string into an elliptic curve point, *Q_ID_*, by way of a specialized hash function.

To encrypt the message, the sender will use a symmetric-key encryption algorithm with a key established using the pairing scheme. In particular, the sender chooses a random integer, *r*, and sends *rP* along with the encrypted message. The symmetric key used to encrypt the message is derived from *e*(*sP, Q_ID_*)*^r^*.

**Private Key Generation**: The private key is derived from the PKG’s secret, *s*, and has the value *sQ_ID_*.
**Decryption**: The recipient uses *rP* to derive the symmetric key needed to decrypt the rest of the message. The key is derived from *e*(*rP, sQ_ID_*). The reader may verify that this quantity is equal to the value, *e*(*sP, Q_ID_*)*^r^*, that the sender used to derive the symmetric key.

In addition to being functional in the sense that the recipient can in fact decrypt messages that are sent to him or her, the above scheme must also be secure, in that 1) no party other than the PKG can compute the recipient’s private key and 2) no party other than the sender can decrypt messages intended for the recipient, without the recipient’s private key. Thus, the security relies on three assumptions:

1. The random oracle assumption – In particular, it is assumed that knowing (or even choosing) the recipient’s ID string will not allow an attacker to find a discrete log for *Q_ID_* (i.e., it should be difficult to find *t* such that *Q_ID_ = tP*.)
2. The CDHP – It is assumed to be difficult to compute *stP* from *sP*, *tP*, and *P*, which implies that it will be hard to compute the private key *sQ_ID_* from the system parameters *sP* and *P* as well as the publicly known hash of the recipient’s identity string, *Q_ID_*. (While the discrete log, *t*, of *Q_ID_* is not known to any party, *Q_ID_* does have a discrete log, and may therefore be written as *tP*, which demonstrates that finding *sQ_ID_* from publically known information is an instance of the CDHP.)
3. The bilinear Diffie-Hellman problem (BDHP) – It is assumed to be difficult to compute *e*(*P,P*)*^rst^* from *rP*, *sP*, *tP*, and *P*. Again, making the substitution of *tP* for *Q_ID_*, we see that finding the decryption key for a message from publicly available information is an instance of the bilinear Diffie-Hellman problem.

The actual Boneh-Franklin scheme is slightly more complicated than the scheme described above. This additional structure allows for a more rigorous proof of security, but the assumptions are essentially the same as the ones stated above. The performance of the scheme is dominated by the cost of the pairing operations. One pairing operation is done by the sender, and one is done by the recipient.

Another important IBE scheme is the Boneh-Boyen [7] scheme. The primary advantages are that the Boneh-Boyen scheme does not require the random oracle assumption in its security proof, and that the sender does not have to perform a bilinear pairing operation in order to encrypt a message. This reduced computation for the sender comes at the cost an extra pairing operation for the recipient, although the recipient can use a mathematical trick to compute the key (which is the ratio of the results of two different pairing operations) more efficiently than he or she could by computing the two pairing operations separately. Since this scheme is somewhat more complicated and non-intuitive than the Boneh-Franklin scheme, it will not be described here, even in simplified form. It works on similar principles, and aside from the random oracle assumption, makes similar security assumptions to the Boneh-Franklin scheme.

A third important IBE scheme is the Sakai-Kasahara [8] scheme. This scheme has been proven secure in the random-oracle model and has better performance overall than either the Boneh-Franklin or the Boneh-Boyen schemes. It does not require a pairing operation for encryption, and only requires a single pairing computation for decryption.

A general advantage of IBE is that it simplifies key management procedures of certificate-based public key infrastructures. IBE also offers interesting features arising from the possibility of encoding additional information into a user’s identity. For example, the inclusion of a date to the ID field can be used to support revocation without the use of certificate revocation lists (CRLs). We note that key escrow is implicit in IBE, which may be seen as an advantage or as a drawback, depending on the situation.

There are two main drawbacks to IBE. First, the PKG has a master secret key, which if compromised would allow an attacker to decipher any message from any user. Second, the security of IBE relies on problems that have not been studied as extensively as the problems that underlie more traditional cryptosystems.

## 4. Other Pairing-Based Schemes

The applications of pairing-based cryptographic schemes go beyond IBE. In this section we give a brief summary of how this technology can be used in other applications. Specifically, we will provide details on a scheme for short digital signatures and a key encapsulation mechanism.

The following (simplified) signature scheme is described in [2]. The signatures produced by the BLS scheme are the shortest of any scheme based on standard assumptions. The signatures are about half the length of those produced by other schemes with the same level of security.

**Setup**: The system parameters require a pairing-friendly elliptic curve for which we have a pairing *e*, as well as a point *P* on the curve. We also require a hash function *H* that outputs points on the elliptic curve.
**Key generation**: The signer selects a secret integer *x*. The public key is the point *K_pub_* = *xP*.
**Signing**: To sign a message *M*, the signer computes the point *σ* = *xH*(*M*).
**Verification**: To verify a signature, the verifier computes *e*(*σ*, *P*) and *e*(*H*(*M*), *K_pub_*) and checks that they are equal.

The scheme is provably secure under the CDHP, assuming that the hash function *H* behaves like a random oracle. An interesting feature is that multiple signatures can be verified in one check.

We also note that the BLS scheme can be turned into an identity-based signature scheme, where the public key *K_pub_* would be derived from a user’s identity. Another well-known identity-based signature scheme is that of Sakai and Kasahara [9]. The advantages of identity-based signature schemes are similar to those of IBE.

Public key cryptography is typically used to establish a shared key between users. With this shared key, users can encrypt and decrypt much more efficiently than with public key cryptography. To simplify key management, an identity-based key encapsulation mechanism (KEM) was proposed by Sakai and Kasahara [9]. A KEM is an algorithm to generate a data-encryption key *k* and then encapsulate *k* by encryption. It is supposed to be followed by a data encryption mechanism (DEM) using *k* as an encryption key. We briefly describe how this can be done:

**Setup**: The PKG chooses a pairing-friendly curve, a pairing *e*, and a base-point *P*. It then generates a secret integer, *s*. The system parameters are *P*, *sP*, *e*, an elliptic curve, and hash functions, *H* and *H*_1_.
**Private Key Generation**: The PKG uses *H*_1_ to convert a user’s ID into an integer *i*. The private key is then the point 1/(*s* + *i*)*P*. The private key is sent to the user.
**Encapsulation**: Let *k* be the desired data-encryption key. A user selects a random integer *r*, and computes the point *U* = *r*(*iP* + *sP*), and the value *V* = *k* ⊕ *H*(*e*(*P*, *P*)*^r^*). He sends the pair (*U*, *V*).
**Decapsulation**: The recipient of the encapsulated data-encryption key first obtains their own private key *T* from the PKG. He then can compute *k* = *V* ⊕ *H*(*e*(*U*, *T*)).

This Sakai-Kasahara KEM is flexible, efficient, and proven secure assuming a variant of the BDHP.

There are many other applications using pairings that have been proposed by researchers. We end this section with a short list of some of these applications. For most of these applications, there are both ID-based and standard (i.e. non ID-based) versions.

- Signcryption – signcryption is the simultaneous operation of signing and encrypting;
- Hierarchical encryption and signatures – allows root PKG to distribute its workload by delegating private key generation and identity authentication to lower-level PKGs;
- Searchable encryption – enables testing whether a given keyword is present in an encrypted message without learning anything about the message;
- Threshold signature schemes – a valid signature can be created only if at least *t* signers cooperate;
- Aggregate signatures – multiple signatures can be aggregated into one compact signature;
- Chameleon hashes and signatures – chameleon hashes are collision resistant functions with a trapdoor for finding collisions. Chameleon signatures are non-repudiable and non-transferrable;
- Blind signatures – enables a user to get a signature from a signer so that the signer does not learn anything about the message being signed;
- Ring signatures – allows any member of a group to sign a message, without revealing which member was the signer;
- Group signatures – similar to ring signatures, but with the inclusion of a “group manager”, who can determine which member signed a message.

## 5. Pairings in Standards

Three organizations have been working on standardization of the pairing-based cryptography schemes. These organizations are the IEEE, IETF, and ISO. This section summarizes the standards activities on pairing-based cryptography, as of 2012.

### IEEE P1363

The working group IEEE P1363 has been active for almost two decades. It was among the first standards groups to standardize public key cryptography schemes, such as RSA encryption and signatures, as well as the Diffie-Hellman and MQV key agreement schemes (over finite fields and elliptic curves). On the other hand, the standards developed in IEEE P1363 are reference standards. That is, they are not application specific and mainly focus on mathematical operations. Furthermore, the schemes are selected from a pool of submissions.

The pairing-based cryptography Project Authorization Request (PAR) was approved in 2005. P1363 announced its call for submissions in 2006. The standard was numbered as IEEE P1363.3. The latest draft [10] was dated September 21, 2011 with a very recent update to Annex A – Number Theoretic Background.

The title of the standard is “IEEE P1363.3: Identity-Based Public-key Cryptography Using Pairings.” It specifies schemes for identity-based encryption, identity-based digital signatures, identity-based signcryption, and identity-based key establishment. All of the schemes use pairings. Short signatures are not included.

The encryption schemes specified in IEEE P1363.3 include:

- SK-KEM: Sakai-Kasahara Key Encapsulation Mechanism (see Sec. 4)
- BF encryption scheme (see Sec. 3)
- BB encryption scheme (see Sec. 3)
- BB-KEM: Similar to BB with key generation. The KEMs include key generation as a part of the scheme.

HIBS (Hierarchical Identity-Based Signature) schemes were submitted, but not selected. However, the HIBS primitives are included in P1363.

Only one signature scheme is specified in P1363.3: the BLMQ signature scheme. This is essentially the Sakai-Kasahara signature scheme mentioned in Sec. 4.

Annex A of P1363.3 specifies pairing-friendly curves such as supersingular curves with embedding degrees 2, 4, and 6, Weierstrass curves including Miyaji, Nakabayashi, and Takano (MNT) curves, Cocks-Pinch curves, Barreto-Naehrig (BN) curves, and KSS curves. The algorithms to produce the above curves are included.

The pairing schemes specified in Annex A of IEEE P1363.3 are the Weil, Tate, Eta, Ate, and R-Ate pairings. The algorithms for computing these pairings are provided. A table is included to indicate which pairing is friendly with which kind of curve.

In summary, IEEE P1363.3 provides sufficient mathematical tools for implementing pairing-based cryptography schemes. However, after six years, the draft IEEE P1363.3 still needs improvements and is not finished. One reason might be a lack of deep mathematical expertise. On the other hand, if the standardization of these schemes were highly demanded or they had been implemented by many vendors, then IEEE P1363.3 would likely have attracted more resources to move it more quickly towards publication.

### IETF

In the IETF, IBE was specified by the working group S/MIME – Mail Security. The specification consists of three RFCs:

- RFC 5091 Identity-Based Cryptography Standard (IBCS) #1: Supersingular Curve Implementations of BF and BB1 Cryptosystems – 2007 [11]
- RFC 5408 Identity-Based Encryption Architecture and Supporting Data Structure – 2009 [12]
- RFC 5409 Using Boneh-Franklin and Boneh-Boyen Identity-Based Encryption Algorithms with the Cryptography Message Syntax (CMS) – 2009 [13].

All three RFCs were authored by Voltage Security, Inc. IBE is used for email encryption in KEM-DEM mode. That is, IBE is used to encrypt a data key which is then used to encrypt data.

E-mail encryption has been a major use case for IBE. It allows a sender to send an encrypted e-mail message without the recipient’s involvement. Note that with today’s virtual private network (VPN) solution, encryption is only applied between a client and an e-mail server. It does not provide end-to-end e-mail encryption between a sender and a recipient. IBE solutions do allow pairwise e-mail encryption.

According to the specifications in these RFCs, the public parameters are provided by a Public Parameter Server (PPS). The interface between a user (a sender or a recipient) and a PPS is assumed to be protected by transport layer security (TLS) [11]. Therefore, the regular PKI is still needed to authenticate the provider for the public parameters. The sender generates a public key using the ID of the recipient and the public parameters. The private key for the recipient is generated by a PKG. It is also assumed that the interface between a recipient and a PKG is protected by TLS. Therefore, client authentication is necessary, which may need to use other means such as passwords, symmetric keys, or even public key certificates for client authentication. Furthermore, the security of IBE for e-mails depends on the TLS tunnel, which provides confidentiality.

The enterprise may be an appropriate place to launch IBE for e-mail encryption. On the other hand, for enterprise e-mail applications, VPN has been launched for remote users to access their e-mails. If IBE were used, then an added benefit would be that it can protect e-mail transmissions between different users, which may not be necessary and is not a common practice.

One possibility is for public e-mail providers such as yahoo or gmail to introduce IBE for e-mail encryption. This would require a standard interface with a PPS and PKG through TLS. We note that key escrow with an e-mail service provider may not be an acceptable feature for users who encrypt e-mails.

The schemes specified by the IETF only use supersingular curves. When these RFCs were developed, the research on pairings over other curves were not well known. RFC 5901 provides a mapping between NIST SP 800-57 security levels and sizes for supersingular curves. The parameter sizes are comparable to RSA as well as DSA and Diffie-Hellman over finite fields, but not DSA and Diffie-Hellman over elliptic curves.

There is a major new standards activity in the IETF on pairing-based cryptography to use IBE for key management. The proposed draft specifies Sakai-Kasahara Key Establishment (SAKKE)[14]. The IBE scheme is used to conduct key transport, which is “drawn” from SK-KEM as defined in P1363.3. The draft was submitted to the IETF to support group key distribution.

Another activity in the IETF was initiated purely for 3GPP standard usage: MIKEY-IBAKE: Identity-Based Mode of Key Distribution in Multimedia Internet Keying (MIKEY). The RFC was published in June 2011 as RFC 6267 [15]. The scheme specified in the RFC uses IBE to encrypt a (regular ECC-based) Diffie-Hellman key exchange. The PKG would not be able to access the key established through a Diffie-Hellman key exchange. Therefore, the IBE scheme is essentially used to authenticate the protocol. The security and robustness need to be further analyzed.

A discontinued draft in S/MIME, regarding a private key request protocol, is worth mentioning. Voltage authored the internet draft, but it was dropped in 2006 (draft-ietf-smime-ibepkg-00). It is unclear why the task was discontinued. So far, no protocol for private key requests has been standardized.

The standardization of pairing-based cryptography schemes in the IETF may lead to an actual application in the Internet environment. However, the slow pace in updating S/MIME RFCs may be a sign of lower demand. Indeed, with the trend to web-based email, even in an enterprise environment, at the expense of traditional POP/IMAP clients, S/MIME may be a fading technology.

### ISO

Considering that the ISO has developed an extremely large portfolio of cryptography schemes, it is not surprising to find some activities on standardizing pairing-based cryptography schemes.

ISO/IEC 15946, “Cryptography Techniques Based on Elliptic Curves Part 1 – General” [16] specified algorithms to compute pairings, including the Weil Pairing and the Tate Pairing.

A study period for identity-based cryptosystems was proposed by Japan, Singapore, and the United Kingdom at the April 2011 meeting in Working Group 2.

The progress on standardizing the pairing-based cryptosystems will depend upon the devoted resources from all the stakeholders. However, the impact on the application of pairing-based cryptography is not expected to be significant.

### Summary

From what we have seen above, pairing-based schemes have been standardized by different organizations. Pairing-based cryptography does not seem to be highly demanded for general usage, based on the observations of P1363.3. IBE in e-mail application as specified in the S/MIME working group of IETF is well standardized but may not be widely used for the reasons discussed above. ISO will standardize pairing-based cryptosystems, but its impact will likely be limited. However, these standards activities are good exercises for the implementation of pairing-based cryptosystems. Once there is a need for an application, the existing tools can be used to implement any of the schemes.

Some of the standards are for very specific applications. Others are too general to provide clear guidance for protocol designers to make a selection. If pairing-based technologies will be introduced for US government needs, then more comprehensive security guidance will be needed to match the security provided through NIST cryptographic toolkits. Furthermore, the infrastructure protocols shall be standardized to communicate with PKGs for interoperability.

## 6. Application Scenarios

While pairing-based cryptography is still an emerging technology, with active research and development, it is being used in large and small-scale applications. This section will describe some of the applications of pairing-based cryptography that are beginning to be implemented by the private and public sectors.

### Identity-Based Encryption

The primary deployed application of pairing-based cryptography is IBE. The largest use case for IBE is e-mail encryption. IBE e-mail encryption software is sold by two major vendors: Voltage Security and Trend Micro.

Unlike S/MIME e-mail encryption using traditional public key cryptography, IBE encrypted e-mail does not require the recipient to pre-enroll. In addition, because IBE keys trace back to the public and private parameters of an organization, it allows easier key management.

On their webpage (as of 2014), Voltage Security reports over 100 million users of IBE encrypted email, primarily in the health IT and insurance sectors.

Besides IBE, pairings have been proven to be useful in a number of areas of cryptography, helping to solve problems that are impossible, difficult, or inefficient with traditional public-key cryptography or symmetric encryption. We mention a few of these applications.

### Enhanced Privacy ID

Enhanced Privacy ID (EPID) is a special group digital signature scheme developed by Intel [17]. It is designed to allow hardware devices to authenticate to a remote system, while preserving the privacy of the device owner. For example, EPID could be used with a hardware cryptographic module, like a Trusted Platform Module (TPM). EPID allows a computer to anonymously prove its possession of a trusted platform module, without revealing information about which TPM module it is, or who the device owner is. In addition to providing anonymity and unlinkability, EPID also allows for the revocation of a signature that is believed to be fraudulent or of a private key that has been compromised.

### Attribute-Based Encryption

In attribute-based encryption (ABE) schemes, an individual can encrypt a message in such a manner that anyone possessing a particular set of attributes, which can be defined at the time of encryption, could decrypt the message. These schemes were pioneered by Sahai and Waters [18]. While it is possible to create similar schemes using symmetric cryptography and traditional public key cryptography, pairing-based approaches require less complicated key management practices.

### Emerging Technologies

Bilinear pairings are being considered for implementing new functionalities. There are many such proposals. We mention only two: “Functional Encryption” uses pairings to construct decryption keys that map ciphertext to an arbitrary function of the plaintext. In this way, a key-generation center can enforce a policy of selective disclosure of plaintext according to attributes of both the ciphertext and the entity requesting a decryption key.

Another example of pairings use is “Searchable Encryption.” This refers to various techniques that allow searching an encrypted database without having to decrypt the database. This might eventually prove very important in securing medical databases.

Bilinear pairings are also being considered for alternative implementations of available functionalities. These include various authentication schemes, privacy-preserving auctions, privacy-friendly aggregation for the smart grid, network communication resilient to traffic analysis, anonymous credentials, and many more.

## 7. Testing Considerations

There are several challenges to developing effective validation testing of any pairing-based technology that NIST might add to its suite of Approved algorithms. The testing will be performed by the independent accredited private-sector laboratories and will be monitored by the Cryptographic Algorithm Validation Program (CAVP) and the Cryptographic Module Validation Program (CMVP) at NIST.

The testing laboratories, by the current accreditation rules, are not expected to have skilled mathematicians on their staffs. The tests that they perform usually consist of feeding some input into an algorithm’s implementation and checking that the expected output value was indeed computed. Therefore, when choosing a pairing-based algorithm, NIST should have a clear vision how the algorithm can be tested under these limitations. This test requirement applies to key generation, elliptic curve computations, checking a curve for its required properties and so on.

We strongly recommend that NIST standardize a list of curves (supersingular curves, or other curves with a low embedding degree) that the implementers will use. At the minimum, each cryptographic module will need to support one of these NIST-recommended curves.

The role of the trusted authority (i.e., the PPS or PKG for IBE) should be considered. The problem is that the CAVP and CMVP test one module at a time. They do not perform any system testing. Since the trusted authority normally resides outside the cryptographic module, this authority cannot be tested together with the module, even if such testing is necessary to assure the module’s compliance with certain requirements of pairing-based cryptography.

The CMVP works closely with the users, vendors and testers of cryptographic modules. Testing pairing based cryptographic schemes will demand implementing new mathematical operations (such as pairings). This process might take a long time and consume many resources. Obviously NIST needs to allocate resources carefully, focusing on the most well launched cryptographic schemes. Therefore, in making the decision to include pairing based schemes into our toolkit, the scale of the potential implementations of the pairing based schemes must be considered.

## 8. Conclusions

As we have seen, pairing-based cryptography has much to offer. Pairing-based schemes, such as IBE, provide special properties which cannot be provided through traditional PKI in a straightforward way. Therefore, pairing-based cryptographic schemes would make a nice addition to NIST’s cryptographic toolkit.

In particular, we have focused attention on IBE. IBE simplifies key management procedures of certificate-based public key infrastructures. IBE also offers interesting features arising from the possibility of encoding additional information into a user’s identity. It has been a decade since the first IBE schemes were proposed. These schemes have received sufficient attention from the cryptographic community and no weakness has been identified. IBE is being used commercially, primarily by Voltage Security and Trend Micro. Intel’s EPID scheme is another example of pairings being used commercially.

The security of pairing-based cryptography is based on the bilinear Diffie-Hellman problem. This is a relatively new problem in cryptography, and has not yet been as well-studied as other problems, such as the DLP or CDHP. Still, since being introduced ten years ago, there have been no breakthroughs in attacks on the BDHP.

Standards bodies have already began standardizing various pairing-based schemes. These include the IEEE, ISO, and IETF. Besides IBE, the standardized schemes include identity-based signatures, identity-based signcyption, identity-based key establishment mechanisms, and identity-based key distribution for use in multimedia (MIKEY-IBAKE).

As a result of our study, we believe there is a good case for allowing government agencies to use pairings. Pairings have been shown to have numerous applications, helping to solve problems that are impossible, difficult, or inefficient with traditional public-key cryptography or symmetric encryption. They also have much potential in areas such as privacy-enhancing technology. We have seen there is a niche for pairing-based schemes, and they will be used in industry regardless of whether they are approved by NIST or not. We therefore recommend NIST standardize IBE and pairing-based cryptography.

One possible approach to standardization would be to include the basic components of pairing-based cryptography in a Federal Information Processing Standard (FIPS). For example, it would contain the NIST-recommended pairing-friendly curves, as well as descriptions of how to compute pairings on them. Then each pairing-based scheme could be described in a Special Publication (SP). Some examples of potential schemes would be the major IBE schemes ([1], [7], [8]), as well as key encapsulation mechanisms, or ID-based signature schemes.

We recognize there are various challenges to standardization by NIST. To implement pairings for any protocol, one must first select an elliptic curve and a specific pairing to use. While there are many options, there is no clear consensus as to which are best to use in practice. Before NIST could recommend which curves and pairings to use, more study is needed. It is also not clear which cryptographic schemes should be included, besides IBE. We are also aware of the challenge in developing a CAVP/CMVP test bed for pairing-based technologies. To address these impediments, further study is needed on the following issues:

- Beyond IBE, which schemes (if any) should we focus on?
- How should specific pairing-friendly elliptic curves be chosen?
- Which pairings should be used in the implementation of these schemes?

We welcome any feedback on our report.

## 9. Appendix

In this appendix, we include more detailed information on pairings. We hope the presentation is easy to understand, rather than being completely formal and rigorous. It is assumed the reader is familiar with elliptic curves and finite fields.

Let *E* be an elliptic curve defined over a finite field *F_q_*. Let *P* and *Q* be points of order *r* on *E*. The point *Q* may possibly be defined over an extension field of *F_q_*. Let *µ_r_* be the group of *r*^th^ roots of unity in *F_q_^k^*. That is, *μ_r_* = {*α* ϵ *F_q_^k^* : *α^r^* = 1} Then a pairing is a map *e* : 〈*P*〉 × 〈*Q*〉 → *µ_r_*. Informally, a pairing takes a pair of points on *E* into a finite field. The pairings used for cryptography must satisfy three properties:

1. The pairing must be *bilinear*: $\begin{array}{l} e\left(P_{1}\;+P_{2},Q\right)=e\left(P_{1},Q\right)e(P_{2},Q)\\ e\left(P,Q_{1}+Q_{2}\right)=e\left(P,Q_{1}\right)e(P,Q_{2}) \end{array}$
2. The pairing must be non-degenerate, which basically means the pairing is not trivial. This is true if *e*(*P*,*Q*) ≠ 1, for some *P*,*Q*.
3. The pairing must be efficiently computable.

The embedding degree *k* is the smallest integer such that *r* | (*q^k^* − 1). Alternatively, *k* is the order of *q* mod *r*. The value of the pairing is an element of the finite field *F_q_^k^*. In order for the pairing to be efficiently computable, *k* must be “small”, certainly less than 100. However, it has been shown that for a randomly chosen elliptic curve, *k* will be roughly the size of *r*, which is at least 160 bits.

Let *ρ* = log*q*/log*r*. For efficient arithmetic, it is desired to have *ρ* as small as possible. We also want *k* small for faster computation of the pairing. To maintain equivalent levels of security, the value of *ρk* should remain constant. Many experts recommend choosing *ρ* ≈ 1, although for some protocols *ρ* ≈ 2 is recommended. Choosing an *r* with low hamming weight is easier if *ρ* ≈ 2. A low hamming weight leads to more efficient computation.

**Table A-1:** Recommended security levels

Security level	*r*	*q^k^*	*k* with *ρ* ≈ 1	*k* with *ρ* ≈ 2
80 bits	160 bits	960–1280 bits	6–8	2–4
128 bits	256 bits	3000–5000 bits	12–20	6–10
256 bits	512 bits	14000–18000 bits	28–36	14–18

An elliptic curve is known as a *pairing-friendly* curve if the following conditions hold:

1. $r\geq \sqrt{q}$ with r | #*E*(*F_q_*) (so the DLP is hard)
2. Embedding degree *k* satisfies $k\leq \frac{\log r}{8}$ (so *k* is small).

Elliptic curves are traditionally written using the Weierstrass equation: *y*^2^ = *x*^3^ + *ax* + *b*. Recently there has been research into computing pairings on other models of elliptic curves. These include:

- Huff curves: *x*(*ay*^2^ − 1) = *y*(*bx*^2^ − 1)
- Jacobi quartics: *y*^2^ = *ex*^4^ − 2*dx*^2^ + 1
- (twisted) Edwards curves: (*a*)*x*^2^ + *y*^2^ = 1 + *dx*^2^*y*^2^

We include a table comparing some of their pairing computation costs.

**Table A-2:** Pairing computation costs

Model	Doubling	Addition	Mixed addition
Huff	1M+1S+(k+11)m+6s	1M+(k+15)m	1M+(k+13)m
Jacobi quartics	1M+1S+(k+9)m+8s+1c		1M+(k+16)m+1s
Edwards	1M+1S+(k+6)m+5s+1c	1M+(k+14)m+1c	1M+(k+12)m+1c
Weierstrass	1M+1S+(k+1)m+11s+1c		1M+(k+6)m+6s

M,S are operations in *F_q_^k^*, while m,s,c denote operations in *F_q_*. M and m are for multiplication, S and s for squaring, and c for multiplication by a constant.

We note there are other models of elliptic curves, as well as hyperelliptic curves. However, for various reasons they are not viable candidates for pairing-based cryptography. We also note that the only known techniques to produce elliptic curves with small *k* all use the Weierstrass form. We now review some of these techniques. A comprehensive survey of all known techniques can be found in [5].

The first example of pairing-friendly curves are *supersingular* curves. A supersingular elliptic curve over *F_p_* is a curve with *p* + 1 points. It has been proven that supersingular curves always have embedding degree *k* ≤ 6. Voltage recommends using the supersingular curve *y*^2^ = *x*^3^ + *b* over *F_p_*, where *p* is a prime with *p* ≡ 11mod12. There has been some concern about the security of using supersingular curves. This arises because the MOV attack uses pairings to make the DLP easier to solve on supersingular curves. However, if parameters are chosen as in Table A-1, then appropriate security can be maintained (according to current knowledge).

Curves which are not *supersingular* are called *ordinary*. With overwhelming probability, a randomly chosen elliptic curve will be ordinary. There are various families of pairing-friendly ordinary curves, all of which are constructed by using what is known as the *complex multiplication* (CM) method. Let *N* be the number of points on *E*, and let *t* = *q* + 1 −*N*. By Hasse’s theorem, we know $t\leq 2\sqrt{q}$. Let *D* = 4*q* − *t*^2^. Informally, the CM method consists of the following steps:

- Construct Hilbert class polynomial *H_D_*(*x*) (possible for *D* < 10^13^);
- Find a root *j* (mod *p*) of *H_D_*(*x*);
- Create an elliptic curve *E* with *j*-invariant *j*;
- Check *E* and its twist *E*′ for a point of large prime order *r.*

The trick to using the CM method is how to find values for the parameters *q*, *t, D*, and *r* so that *k* is small.

There are many families of ordinary curves given in [5]. The Cocks-Pinch family consists of curves where *k* can be arbitrary, and *ρ* ≈ 2. MNT curves are ordinary curves with *k* = 3, 4, or 6 and *ρ* ≈ 1. Some of the fastest timings for pairings use BN curves, which have *k* = 12 and *ρ* ≈ 1. Other families include Freeman curves, BLS curves, BW curves, and Dupont/Enge curves, just to name a few. Finding pairing-friendly curves is an active area of research.

We now turn to the various known pairings, and how to compute them. The two most well-known pairings are the Weil and Tate pairings. Researchers have found several other pairings as they have tried to optimize computation of these pairings. These include the reduced Tate, Eta, generalized Eta, Ate, twisted Ate, R-Ate pairings.

Let *E*[*r*] denote the set of all points of *E* which have *r*-torsion. As an abstract group, *E*[*r*] is the product of two cyclic groups of order *r*, or E[r]≅ZrZ×ZrZ. We choose *P* and *Q* to be generators for this group. Recall the Frobenius map on *E* is *π*(*x*, *y*) = (*x^q^*, *y^q^*). In pratice, *P* and *Q* are chosen so that *π*(*P*) = *P*, *π*(*Q*) = *qQ*. This makes *P* ∈ *E*(*F_q_*) and *Q* ∈ *E*(*F_q_^k^*).

A divisor is a formal finite sum $D=\sum a_{j}[P_{j}]$, where *a_j_* ∈ *Z*, and *P_j_* are points on *E.* Let $\deg(D)=\sum a_{j}$ and $\text{sum}(D)=\sum a_{j}P_{j}$, which is a point on *E*. Let $f(x,y)=\frac{r(x,y)}{s(x,y)}$ be a function on *E*. Functions over an elliptic curve can be written in more than one way. For example, $f(x,y):=\frac{x}{y}=\frac{y}{x^{2}-1}$ on the curve *y*^2^ = *x*^3^ − *x*. We define zeroes and poles of a function *f* in the following way. The point *P* is a zero of *f* if *r*(*P*) = 0, and *s*(*P*) ≠ 0, while *P* is a pole of *f* if *s*(*P*) = 0, and *r*(*P*) ≠ 0. It is known that there are only finitely many zeros and poles of a function *f*, and that the total number of zeroes is equal to the total number of poles (counting multiplicities).

We define the divisor of *f* to be

$$\text{div}(f)=\sum_{P\in E}ord_{P}(f)[P]=\sum_{zeros}ord_{P}(f)[P]-\sum_{poles}ord_{P}(f)[P]$$

For example, suppose *P*_1_, *P*_2_, *P*_3_ are on *E* and also on the line *ax + by + c* = 0. Then div(*ax + by + c*) = [*P*_1_] + [*P*_2_] + [*P*_3_] – 3[∞].

All known pairings are computed using *Miller functions*. A Miller function is denoted *f_i,P_*, which is such that div(*f_i,P_*) = *i*[*P*] − [*iP*] − (*i* − 1)[∞]. Note that div(*f_r,P_*) = *r*[*P*] − *r*[∞]. We now show how the various pairings are computed (assuming *k* > 1), as well as their domain.

**Table A-3:** Pairings

Pairing	Definition	Domain
Weil	(−1)*^r^f_r_*,*_P_*(*Q*)/*f_r_*,*_Q_*(*P*)	E[*r*] × E[*r*]
Tate	*f _r_*,*_P_*(*Q*)	<*P*> × E/*r*E
Reduced Tate	*f _r_*,*_P_*(*Q*)^n^	<*P*> × <*Q*>
Eta	*f*_λ_,*_P_*(*Q*)^n^	<*P*> × <*Q*>
Ate	*f_t_*_−1_,*_Q_*(*P*)*^n^*	<*Q*> × <*P*>
Generalized Eta	*f_λ_^e^*,*_P_*(*Q*)*^n^*	<*P*> × <*Q*>
Twisted Ate	*f*_(_*_t_*_−1)_*^e^*, *_Q_*(*P*)^n^	<*P*> × <*Q*>

Explanation: *t* is the trace, i.e. #*E*(*F_q_*) = *q* + 1 − *t*, <*P*> = *E*(*F_q_*)[*r*]

*λ* = (*t* − 1)*^k/d^*, where *d* = degree of *E*^twist^,    <*Q*> ⊆ *E*(*F_q_^k^*)[*r*]

*k* is the embedding degree, Frob(*P*) = *P*   Frob(*Q*) = *qQ*

$n=\frac{q^{k}-1}{r}$ *e* is certain integer mod *r*.

If *k* = 1, then replace *Q* by divisor *D* = [*Q* + *R*] − [*R*].

To compute the functions *f_i_*,*_P_*, we use Miller’s algorithm. This algorithm relies on the result that $f_{i+j,P}=f_{i,P}f_{j,P}\frac{l_{iP,jP}}{v_{(i+j)P}}$, where *l_R_*,*_S_* is the line through *R* and *S*, and *v_R_* is the vertical line through *R*.

### Miller’s Algorithm

**Table:**

Input: *P*,*Q*, and integer *i*
Output: *f_i_*,*_P_*(*Q*)
1. B = Bits(*i*), *T* = *P*, *f* = 1
2. For *j* from #B-1 to 1 do
3. *l* = tangent line through *T*, *v* = vertical line through *T*
4. $f=f^{2}\frac{l(Q)}{v(Q)}$
5. *T* = 2*T*
6. If B[*j*] = 1 then do
7. *l* = line through *T* and *P, v* = vertical line through *T* + *P*
8. $f=f\frac{l(Q)}{v(Q)}$
9. *T* = *T* + *P*
10. Return *f*

There are various speed-ups researchers have discovered to optimize computation of these pairings. If the embedding degree is divisible by *d* = 2, 3, 4, or 6 then *twisting* can be used for point compression. This allows working in the finite field *F_q_^k^*^/^*^d^*, instead of *F_q_^k^* Some other tricks involve reducing the number of iterations in Miller’s algorithm, eliminating denominators in Miller’s algorithm, and making the final exponentiation easier to evaluate.

In terms of speed, the Weil pairing is generally regarded as the slowest, but it does have some properties like *e*(*P*, *P*) = 1, which might be needed. The Tate-variants (eta, ate, R-ate, twisted ate) are usually the quickest as they were designed to require less Miller iterations. The current records for speed use the optimal ate pairing on BN curves. At the 128 bit security level, the pairing takes 0.6 ms on 2.8 GHz PC. We note that the time spent on the Miller loop is approximately same as time spent on the final exponentiation.

There is no single pairing choice that is the all-around best. It depends on the specific protocol, security level needed, curve choice, etc… We now mention several such considerations.

For most protocols, if a formal proof of security is required, then supersingular curves must be used. Supersingular curves also have the advantage of using distortion maps to change the domain from 〈*P*〉 × 〈*Q*〉 to 〈*P*〉 × 〈*P*〉. The Eta pairing can only be defined over supersingular curves.

We categorize pairings into three types. We may write each pairing as *e* : *G*_1_ × *G*_2_ → *G_T_*, where the domain is *G*_1_ × *G*_2_. See Table A-3 for the domains of common pairings. Typically the points in *G*_1_ have coordinates in *F_q_*, while those in *G*_2_ have coordinates in *F_q_^k^*. We say the pairing *e* is Type 1 if *G*_1_ = *G*_2_. If *G*_1_ ≠ *G*_2_, and there is an efficiently computable homomorphism *φ* : *G*_2_ → *G*_1_ (but not vice-versa), then *e* is Type 2. Finally, *e* is Type 3 if *G*_1_ ≠ *G*_2_, and there are no efficient homomorphisms between *G*_1_ and *G*_2_. Pairings on supersingular curves can be implemented as Type-1 pairings.

The following table from [6] illustrates some of the differences between pairings. A checkmark ✓ denotes the pairing can easily achieve the property, while an x denotes it cannot. Let *p* be the characteristic of *F_q_*, i.e., *q* = *p^f^*.

**Table A-4:** Comparison of pairings [6]

Type	Hash to *G*_2_	Short *G*_1_	Homomorphism	Poly time generation
1 (*p* = 2 or 3)	✓	x	✓	x
1 (*p* > 3)	✓	x	✓	✓
2	x	✓	✓	✓
3	✓	✓	x	✓

Hash to *G*_2_: One can hash into *G*_2_
Short *G*_1_: There is a (relatively) short representation for elements of *G*_1_
Homomorphism: There is an efficiently computable *φ* : *G*_2_ → *G*_1_
Poly time generation: One can generate system parameters (including groups and a pairing) achieving at least *K* bits of security in time polynomial in *K*

We also include another table from [6] showing the effect of raising the security level on pairings. The ratings are from zero stars (operation is impossible) to three stars *** (there is a good solution).

**Table A-5:** Efficiency and Bandwidth Comparisons [6]

Type	*K*	H1	H2	S1	S2	E1	E2	E3	P	F
Type 1 (p=2)	80	***	***	**	1	**	1	8/7	***	*
	256	*	*	*	1	*	1	8/7	*	*
Type 1(p=3)	80	***	***	***	1	***	1	3	***	*
	256	*	*	*	1	*	1	3	*	*
Type 1 (p>3)	80	**	**	*	1	*	1	1/4	***	***
	256	*	*	*	1	*	1	1/4	*	***
Type 2	80	***		***	*k*	***	*k*^2^	*k*^2^/16	*	***
	256	**/***		*/***	*k*	**/***	*k*^2^	*k*^2^/16	*	***
Type 3	80	***	*	***	*e*	***	*e*^2^	*k*^2^/16	***	***
	256	**/***	*	*/***	*e*	**/***	*e*^2^	*k*^2^/16	***	***

$$e=\{\begin{array}{l} k/\gcd(k,4)\;\text{if}\;D=-4\\ k/\gcd(k,6)\;\text{if}\;D=-3\;k=\text{embedding degree},D=4q-t^{2}\\ k/\gcd(k,2)\;\text{if}\;D<-4 \end{array}$$

*K*: Security level
H1: Can one hash to *G*_1_ efficiently?
H2: Can one hash to *G*_2_ efficiently?
S1: Is there short representation for elements of *G*_1_? (with security level *K*, elements of *G*_1_ represented with ≤ 2*K* + 10 bits)
S2: Is there short representation for elements of *G*_2_?
E1: Are group operations in *G*_1_ efficient? (with security level *K* is efficiency comparable to usual ECC with same security)?
E2: What is ratio of complexity of group operations in *G*_2_ to complexity of group operations in *G*_1_?
E3: What is the ratio of complexity of group operations in *G_T_* to the complexity of those in *G*_1_?
P: Is the pairing efficient? (meaning how does speed of pairing compare with alternative groups of same security level)
F: Is there wide flexibility in choosing system parameters? (meaning is it necessary that all users to share one curve, or is there plenty of freedom for users to generate their own curve of any desired security level)
